# Discovery of Uracil Derivatives as Potent Inhibitors of Fatty Acid Amide Hydrolase

**DOI:** 10.3390/molecules21020229

**Published:** 2016-02-18

**Authors:** Yan Qiu, Yang Zhang, Yuhang Li, Jie Ren

**Affiliations:** 1 Department of Basic Medical Sciences, Medical College, Xiamen University, Xiamen 361102, China; zhyang880105@163.com (Y.Z.); mfaoxmu@126.com (Y.L.); renjie_7912@xmu.edu.cn (J.R.); 2 Quality Responsibility Pharmaceuticals Co., Ltd., Wuhan Research & Development Center, Wuhan 430075, China

**Keywords:** fatty acid amide hydrolase (FAAH), uracil derivatives, FAAH inhibitor, amidation

## Abstract

Fatty Acid Amide Hydrolase (FAAH) is an intracellular serine enzyme involved in the biological degradation of the fatty acid ethanolamide family of signaling lipids, which exerts neuroprotective, anti-inflammatory, and analgesic properties. In the present study, a conjugated 2,4-dioxo-pyrimidine-1-carboxamide scaffold was confirmed as a novel template for FAAH inhibitors, based on which, a series of analogues had been prepared for an initial structure-activity relationship (SAR) study. Most of the synthesized compounds displayed moderate to significant FAAH inhibitory potency. Among them, compounds **11** and **14** showed better activity than others, with IC_50_ values of 21 and 53 nM. SAR analysis indicated that 2,4-dioxopyrimidine-1-carboxamides represented a novel class of potent inhibitors of FAAH, and substitution at the uracil ring or replacement of the *N*-terminal group might favor the inhibitory potency. Selected compounds of this class may be used as useful parent molecules for further investigation.

## 1. Introduction

Anandamide (AEA) belongs to the endocannabinoid family, a series of endogenous fatty acid ethanolamides (FAEs), which participates in the control of pain [1,2], inflammation [3], and cognition [4,5], through engaging cannabinoid receptors 1 and 2 (CB_1_, CB_2_) [6]. The signaling function of AEA is terminated by enzymatic hydrolysis in processes principally mediated by FAAH, a distinct serine hydrolase [7,8]. Selective FAAH inhibitors have been identified to elevate endogenous AEA levels, which display a more restricted spectrum of pharmacological effects than CB_1_ agonists. Pharmacologic or genetic disruption of FAAH showed antihyperalgesia, antianxiety and antidepression without exhibiting deficits in motility, cognition or body temperature [9,10], which suggests that FAAH is an attractive therapeutic target for treatment of pain, inflammation, anxiety, depression and other CNS disorders.

**Figure 1:**
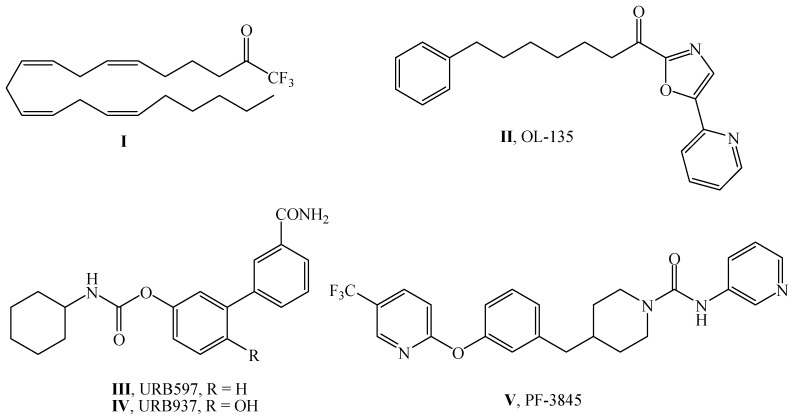
Structures of FAAH inhibitors.

FAAH is a membrane-bound protein belonging to the amidase signature family of serine hydrolases, and is located on the outer face of the endoplasmic reticulum and mitochondria [11]. Its bioactivity is optimal at pH 8.0–9.0. Mutagenesis studies revealed that FAAH possessed a distinct serine-serine-lysine catalytic triad. Among which, Ser241 was served as the nucleophile and Lys142 as a key acid and base of the catalytic cycle [12]. The first-generation FAAH inhibitors such as the substrate analogues **I** [13] were proved invaluable for biochemical and structural studies of FAAH. While, due to the high hydrophobicity and low selectivity, these compounds were not suitable to serve as the templates for new drug development. Several classes of FAAH inhibitors with improved selectivity and *in vivo* activity have been reported in recent years (Figure 1).

One class of FAAH inhibitors is reported as electrophilic ketone compounds, which contains α-ketoheterocycles like **II** (OL-135) to form a reversible hemiacetal bond with the catalytic serine [14]. These compounds displayed excellent *in vivo* potency and selectivity, while, reversible action led to elevated levels of several *N*-acylethanolamine (NAE) substrates, and only transient elevations in AEA. URB597 (**III**) is a representative of carbamate inhibitors of FAAH [9]. It was shown that carbamate inhibitors formed a covalent bond with active site Ser241 nucleophile by carbamylation in an irreversible mechanism [15]. An additional hydroxy substitution to the proximal phenyl ring of URB597 restricted the compound (**IV**, URB937) to cross the blood-brain barrier, avoiding the potential CNS effects [16]. Urea inhibitors were reported since 2006 by some outstanding pharmaceutical companies as Pfizer, Takeda, Johnson & Johnson, Lilly and Astellas [17]. As the most potent inhibitor, PF-3845 (**V**) gains its potency from a more extended set of van der Waals interactions between the biaryl ether piperidine moiety and the hydorphobic acyl chain-binding pocket (ACBP) of FAAH [18].

A computer-aided virtual screening combined with enzyme activity assay on in-house compounds indicated that a known uracil derivative (**1**) exhibited moderate enzyme inhibitory activity (IC_50_ = 0.11 ± 0.03 μM). Although in 2013, uracil derivatives were reported as a novel class of potent inhibitors of Acid ceramidase (AC) [19,20], substituted 2,4-dioxopyrimidine-1-carboxamides were not applied as a template in design and synthesis of FAAH inhibitors. Considering that all of the reported AC inhibitors have a retained C6 aliphatic chain [20], in the present work, we turned to replace it with the more rigid aromatic group class to improve druggable properties. A series of uracil derivatives were then prepared based on the parent molecule **1** as potent enzyme inhibitors, and initial elucidation of the role in FAAH inhibition was performed.

## 2. Results

### 2.1. Chemistry

**Scheme 1:**
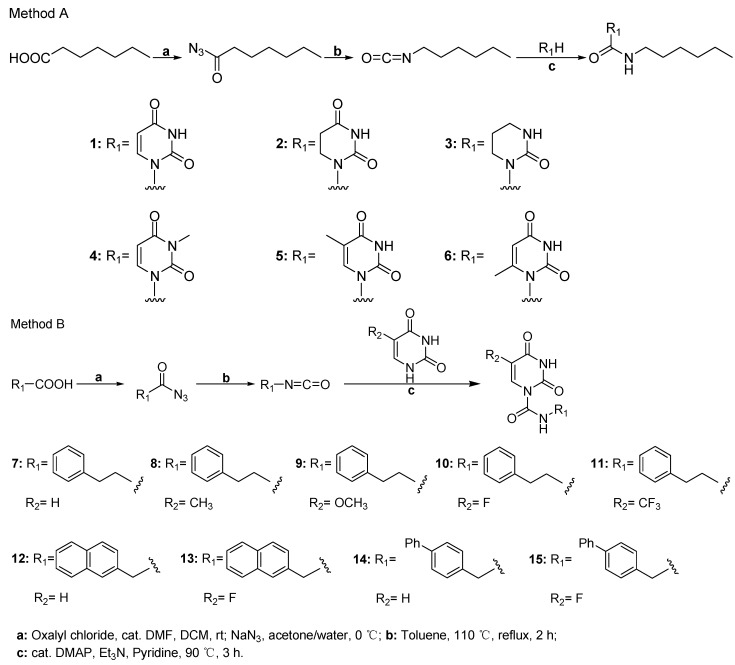
Preparation of compounds **1**–**15**.

Compounds **1**–**15** were prepared by the amidation of uracil derivatives (or their analogues) with the corresponding isocyanates in the presence of DMAP/triethylamine (Scheme 1). The above isocyanates were produced by acyl azides reaction and Curtius rearrangement of the corresponding carboxylic acids (Scheme 1).

**Scheme 2:**
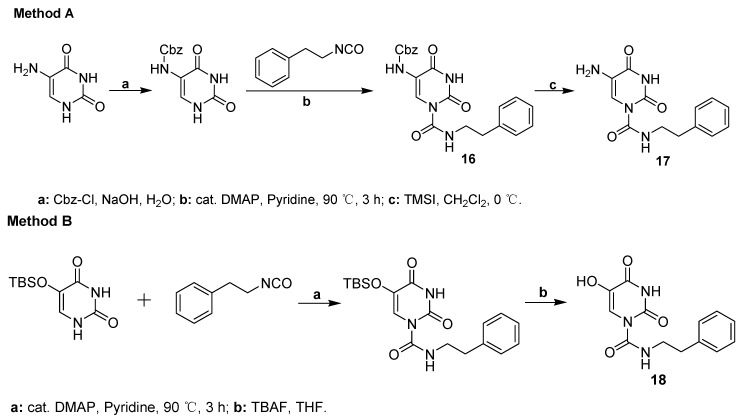
Preparation of compounds **16**–**18**.

The active substituents at position 5 of uracil ring, such as amino or hydroxy, should be protected before amidation. Here, the carboxybenzyl group was used to protect amines, while the *t*-butyl-dimethylsilyl group was used for active hydroxy protection. Compounds **16**–**18** were then synthesized by means of the amidation of protected uracil derivatives with phenylethyl isocyanates in the presence of DMAP/pyridine (Scheme 2), and the protecting groups were removed after amidation reaction.

**Scheme 3:**
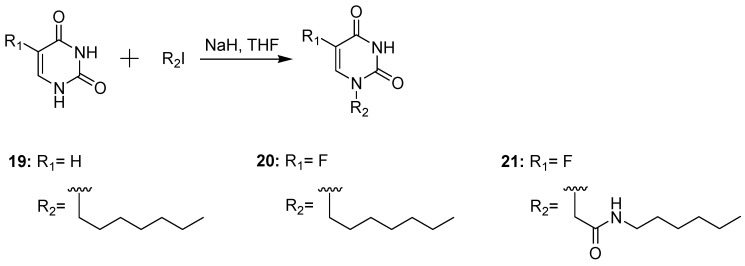
Preparation of compounds **19**–**21**.

Compounds **19**–**21** were prepared by *N*-alkylation reaction of uracil derivatives with the corresponding alkyl iodide in the presence of NaH/THF (Scheme 3).

### 2.2. FAAH Inhibition

The substituted 2,4-dioxopyrimidine-1-carboxamides and the related analogues described above were subjected to *in vitro* biological evaluation toward recombinant rat FAAH derived from HEK293-rFAAH cell. Enzyme assays were performed at 37 °C by incubating 30 µg of rFAAH protein with 25 μM anandamide as substrate in Tris-HCl buffer containing fatty acid-free BSA (0.05%). When the reactions were terminated, 1 nmol heptadecanoic acid (17:0 FFA) was added as an internal standard. URB597 was used as a positive control. The hydrolyzate of AEA (arachidonic acid), together with heptadecanoic acid was detected in HPLC/MS/MS to estimate the potency of rFAAH inhibition according to the methods previously described [21]. IC_50_ values of the above compounds were expressed in Table 1.

## 3. Discussion

The purpose of the present work was to investigate substituted 2,4-dioxo-pyrimidine-1-carboxamides as a potent class of FAAH inhibitors. We firstly examined the role of the uracil scaffold. Compound **2**, the dihydro derivative of **1**, showed no inhibitory activity toward FAAH, which highlighted the important function of the C5–C6 double bond. Replacement of the uracil ring with tetrahydro-2-one-pyrimidine (compound **3**) also led to the loss of FAAH inhibitory activities. We next evaluated the role of the urea linkage. Compared with **1**, when the carboxamide was reduced to methylene as in **19** and **20**, FAAH activity was not inhibited at all. Even when the carboxamide group was retained, destroying of urea linkage by inserting a methylene into the urea bond could lead to a total loss of the FAAH inhibitory activity (**21**, IC_50_ > 100 μM). It was suggested that a complete conjugated 2,4-dioxo-pyrimidine-1-carboxamide system was necessary for enzyme inhibition, which represents a novel template for FAAH inhibitor development and research.

As the flexible aliphatic chain may weaken the prospects of pharmaceutical development of **1** as a FAAH inhibitor, we turned to using the more rigid aromatic group class to replace it. Replacement of the hexyl chain of **1** with a naphthylmethyl group led to a 8-fold decrease in inhibitory activity (**12**, IC_50_ = 0.81 ± 0.10 μM), and substitution with a 2-phenylethyl side chain in the same position caused a slight 3-fold decrease in potency (compound **7** *vs.* **1**). Notably, changing the steric configuration of the lipophilic chain by substitution of 4-biphenylmethyl group resulted in a new double digit nanomolar inhibitor **14** (IC_50_ = 0.053 ± 0.006 μM).

We finally focused our attention on uracil ring substitutions to further investigate the structure-activity relationships. Methylation on unsubstituted positions of the uracil ring of **1** was then explored, which was not beneficial for the activity. The introduction of a methyl group at 3-, or 6-position of the uracil ring led to 8- to 10- fold decrease in potency (**4**, **6** *vs.* **1**). Considering that the 5-methyl derivatives were only 2- to 3- fold less potent than the related parent compounds (**5** *vs.* **1**, **8** *vs.* **7**), additional substitutions were then kept at position 5 for further SAR study.

As illustrated in Table 1, introduction of a methoxy, amino and hydroxy group at 5-position of **7** declined the inhibitory activity toward FAAH, as compound **9**, **17** and **18** yielded IC_50_ values of 0.53, 1.33 and 0.54 μM, respectively. Substitution of the amine group of compound **17** by *N*-benzyloxycarbonyl amino group (**16**) increased the inhibitory potency toward FAAH for about 10 times, which suggested that a free amine group in position 5 of the uracil ring is unfavorable to the activity. Replacing the methyl group of **8** with electron-withdrawing substituents, such as –F and –CF_3_, resulted in two novel double digit nanomolar FAAH inhibitors **10** (IC_50_ = 0.098 ± 0.01 μM) and **11** (IC_50_ = 0.021 ± 0.004 μM), which had a more favorable impact on the inhibitory potency than substitution of electron-donating groups. Meanwhile, fluorine substitution at the uracil ring did not always cause an increase in potency. Replacement of a hydrogen atom with fluorine at 5-position of **12** led to a slightly decrease in potency (**13** *vs.* **12**), and compound **15** showed a 2-fold less potent than **14**, which might be affected by additional features that reduced the binding site affinity.

## 4. Experimental Section

### 4.1. General

In the present study, all chemicals were purchased from Sigma-Aldrich (Shanghai, China), seeking the highest grade commercially available unless otherwise indicated. Some solvents were distilled prior to use, such as methylene chloride (CH_2_Cl_2_), distilled from phosphorus pentoxide, dimethylformamide (DMF), distilled from calcium hydride and tetrahydrofuran (THF), distilled from sodium benzophenone ketyl. Silica gel (300–400 mesh) from Yantai Athy Chemical Technology Co. Ltd. (Zhifu, China) was used for column chromatography. Mixtures of increasing polarity of ethyl acetate and petroleum ether (PE) (60–90 °C) were used as eluents unless otherwise stated. ^1^H-NMR spectra were recorded on a Bruker 400 (400 MHz) spectrometer (Bruker, Fallanden, Switzerland) using CDCl_3_, DMSO-*d*_6_, or CD_3_OD as the solvent with Me_4_Si as the internal standard. ^13^C-NMR spectra were recorded on a Bruker 400 (100 MHz) spectrometer. IR spectra were recorded on a Nicolet AVATAR 360 RT-IR spectrophotometer (Thermo Electron Inc., San Jose, CA, USA). Mass spectra were recorded on an MDS SCIEX 3200Q TRAP mass spectrometry (MS) system (Applied Biosystems/MDS Sciex, Concard, ON, Canada) with electrospray ionization (ESI) and direct injection. Elemental analyses were performed on an Elementar Vario EL analyzer (Vario EL, Elementar Analyser systeme GmbH, Hanau, Germany). HPLC analyses were run on a 1200 series HPLC (Agilent, Shanghai, China) system equipped with a photodiode array detector. PDA range was 210–600 nm. Analyses were performed on a Hypersil Gold C18 column (dimensions 250 × 4.6 mm, particle size 5 μm, Thermo Scientific, Waltham, MA, USA). A constant mobile phase was 85% methanol in H_2_O, and the flow rate was set at 1 mL·min^−1^. All final compounds showed ≥95% purity by HPLC and NMR analysis.

### 4.2. General Procedure for the Synthesis of Uracil Derivatives ***1***–***15***

Compounds **1**–**15** were synthesized according to Scheme 1. The appropriately substituted carboxylic acid (0.5 mmol) was dissolved in dry CH_2_Cl_2_ (5 mL). Oxalyl chloride (0.6 mmol, 0.05 mL) and DMF (0.01 mL) were added, and the reaction mixture was stirred at 0 °C for 1 h. The solvent was evaporated under reduced pressure, keeping the temperature below 30 °C. The crude residue was redissolved with anhydrous acetone (5 mL) and then dropwise added into a stirred and ice-cold solution of NaN_3_ (1.0 mmol, 33 mg) in H_2_O (1 mL). The resulting mixture was stirred at 0 °C for 30 min, and extracted with EtOAc. The organic layer was washed with brine, dried over anhydrous Na_2_SO_4_, and concentrated under reduced pressure. The residue was then dissolved in toluene (15 mL), and refluxed for 2 h. The reaction solution was cooled to room temperature, and dropwise added to a solution of the corresponding uracil derivatives (0.5 mmol) in dry pyridine (0.5 mL). After being stirred at 90 °C for 3 h, the mixture was cooled and evaporated. The crude was purified by flash chromatography on silica gel to afford compounds **1**–**15**.

*N-Hexyl-2,4-dioxo-pyrimidine-1-carboxamide* (**1**). This compound was obtained in 84% yield as a white amorphous powder. Detailed MS, ^1^H- and ^13^C-NMR results were according to the literature data [19].

*N-Hexyl-2,4-dioxotetrahydropyrimidine-1-carboxamide* (**2**). This compound was obtained in 67% yield as a white amorphous powder, Detailed MS, ^1^H- and ^13^C-NMR results matched the literature values [20].

*N-Hexyl-2-oxotetrahydropyrimidine-1-carboxamide* (**3**). This compound was obtained as a white amorphous powder in 74% yield ^1^H-NMR (D_2_O-CDCl_3_) δ 0.86 (t, *J* *=* 6.8 Hz, 3H), 1.23–1.28 (m, 6H), 1.47–1.52 (m, 2H), 1.95–2.00 (m, 2H) 3.24–3.29 (m, 2H), 3.35 (m, 2H), 3.78–3.84 (m, 2H); ^13^C-NMR (CDCl_3_) *δ* 14.3, 21.5, 22.5, 26.3, 29.1, 31.3, 32.5, 40.8, 40.9, 152.0, 161.0.

*N-Hexyl-3-methyl-2,4-dioxopyrimidine-1-carboxamide* (**4**). This compound was obtained as a white amorphous powder in 49% yield, Detailed MS, ^1^H- and ^13^C-NMR results were in accord with the literature [20].

*N-Hexyl-5-methyl-2,4-dioxopyrimidine-1-carboxamide* (**5**). This compound was obtained in 55% yield as a white amorphous powder. Detailed MS, ^1^H- and ^13^C-NMR results were in good accord with the literature [20].

*N-Hexyl-6-methyl-2,4-dioxopyrimidine-1-carboxamide* (**6**). This compound was obtained as a white amorphous powder (63% yield). ^1^H-NMR (CDCl_3_/pyridine-*d*_5_) δ 0.90 (t, *J* *=* 6.8 Hz, 3H), 1.29–1.38 (m, 6H), 1.55–1.65 (m, 2H), 1.76 (s, 3H), 3.34–3.44 (m, 2H), 6.25 (br, 1H), 8.24 (br, 1H), 9.25 (br s, 1H); ^13^C-NMR (CDCl_3_/pyridine-*d*_5_) δ 9.4, 14.0, 22.5, 26.5, 29.2, 31.4, 41.2, 102.5, 145.3, 150.0, 155.1, 163.1; MS (ESI, *m*/*z*): 252 (M − H)^−^.

*N-(2-Phenylethyl)-2,4-dioxopyrimidine-1-carboxamide* (**7**). This compound was obtained as a white amorphous powder (75% yield). IR (ν_max_, cm^−1^): 2950, 2918, 2849, 1725, 1580, 1547, 1402, 1383; ^1^H-NMR (DMSO-*d*_6_) δ 2.84 (t, *J* = 7.0 Hz, 2H), 3.50–3.55 (m, 2H), 5.80 (d, *J* = 8.4 Hz, 1H), 7.22–7.31 (m, 5H), 8.21 (d, *J* = 8.4 Hz, 1H), 9.20 (br, 1H), 11.72 (br, 1H); ^13^C-NMR (DMSO-*d*_6_) δ 35.2, 42.4, 104.1, 126.8, 128.9, 129.2, 139.1, 139.3, 150.4, 152.0, 163.2; MS (ESI, *m*/*z*): 258 (M − H)^−^.

*5-Methyl-N-(2-phenylethyl)-2,4-dioxopyrimidine-1-carboxamide* (**8**). This compound was obtained as white amorphous powder in 70% yield. IR (ν_max_, cm^−1^): 3456, 2950, 2917, 2849, 1580, 1068; ^1^H-NMR (CDCl_3_) δ 1.98 (s, 3H), 2.91 (t, *J* = 7.1 Hz, 2H), 3.62–3.65 (m, 2H), 7.21–7.31 (m, 5H), 8.22 (s, 1H), 9.16 (br, 1H), 9.41 (br, 1H); ^13^C-NMR (CDCl_3_) δ 12.4, 35.5, 42.5, 112.4, 126.7, 128.7, 128.8, 134.5, 138.3, 150.1, 151.5, 163.6; MS (ESI, *m*/*z*): 272 (M − H)^−^.

*5-Methoxy-N-(2-phenylethyl)-2,4-dioxopyrimidine-1-carboxamide* (**9**). This compound was obtained in 65% yield as a white amorphous powder. ^1^H-NMR (DMSO-*d*_6_) δ 2.84 (t, *J* *=* 7.0 Hz, 2H), 3.50–3.55 (m, 2H), 3.68 (s, 3H), 7.22–7.31 (m, 5H), 7.70 (s, 1H), 9.25 (m, 1H), 12.0 (s, 1H); ^13^C-NMR (DMSO-*d*_6_) δ 35.2, 42.4, 56.5, 116.2, 126.8, 128.3, 128.7, 130.2, 136.5, 150.1, 150.3, 158.8; MS (ESI, *m*/*z*): 288 (M − H)^−^.

*5-Fluoro-N-(2-phenylethyl)-2,4-dioxopyrimidine-1-carboxamide* (**10**). This compound was obtained as a white amorphous powder in 39% yield. Detailed MS and ^1^H-NMR results were in agreement with the literature [22].

*N-(2-Phenylethyl)-5-trifluoromethyl-2,4-dioxopyrimidine-1-carboxamide* (**11**). This compound was obtained in 23% as a white amorphous powder. ^1^H-NMR (CDCl_3_) δ 2.76 (t, *J* = 6.9 Hz, 2H), 3.41–3.45 (m, 2H), 7.25–7.35 (m, 5H), 8.93–9.01 (m, 3H); ^13^C-NMR (CDCl_3_) δ 35.4, 42.4, 114.3, 121.2 (q, *J* = 268 Hz), 126.8, 128.9, 129.2, 139.1, 139.7, 149.0, 150.7, 156.8; MS (ESI, *m*/*z*): 326 (M − H)^−^.

*N-(1-Naphthylmethyl)-2,4-dioxopyrimidine-1-carboxamide* (**12**). This compound was obtained as white crystals (20% yield). Mp: 84.0–84.6 °C; IR (ν_max_, cm^−1^): 3426, 2955, 2917, 2849, 1726, 1578, 1380; ^1^H-NMR (DMSO-*d*_6_) δ 4.66 (d, *J* = 5.3 Hz, 2H), 5.81(d, *J* = 8.4 Hz, 1H), 7.50–7.51 (br, 3H), 7.85 (s, 1H), 7.89–7.90 (br, 3H), 8.23 (d, *J* = 8.4 Hz, 1H), 9.67 (br, 1H), 11.77 (br, 1H); ^13^C-NMR (DMSO-*d*_6_) δ 44.6, 104.1, 126.0, 126.3, 126.4, 126.7, 128.0, 128.1, 128.5, 132.7, 133.3, 136.3, 139.3, 150.9, 152.0, 163.3; MS (ESI, *m*/*z*): 294 (M − H)^−^.

*5-Fluoro-N-(1-naphthylmethyl)-2,4-dioxopyrimidine-1-carboxamide* (**13**). This compound was obtained as a white amorphous powder in 27% yield. ^1^H-NMR (DMSO-*d*_6_) δ 4.67 (d, *J* = 5.6 Hz, 2H), 7.49–7.52 (m, 3H), 7.84–7.91 (m, 4H), 8.42 (d, *J* = 7.6 Hz, 1H), 9.67 (t, *J* = 6.0 Hz, 1H), 12.30 (d, *J* = 4.4 Hz, 1H); ^13^C-NMR (DMSO-*d*_6_) δ 44.7, 123.6, 125.4, 126.3, 126.5, 126.7, 127.9, 128.0, 128.5, 132.7, 133.3, 137.5, 140.0, 142.3, 150.6, 157.6; MS (ESI, *m*/*z*): 312 (M − H)^−^.

*N-[2-(4-Biphenyl)methyl]-2,4-dioxopyrimidine-1-carboxamide* (**14**). This compound was obtained in 30% yield as a white amorphous powder, yield 30%. IR (ν_max_, cm^−1^): 3440, 2956, 2917, 2849, 1723, 1578, 1464, 1380; ^1^H-NMR (CDCl_3_) δ 4.61 (d, *J* = 4.6 Hz, 2H), 5.90 (d, *J* = 8.5 Hz, 1H), 7.34–7.44 (m, 5H), 7.57 (br, 4H), 8.28 (br, 1H), 8.43 (d, *J* = 8.5 Hz, 1H), 9.47 (br, 1H); MS (ESI, *m*/*z*): 320 (M − H)^−^.

*N-[2-(4-biphenyl)methyl]-5-fluoro-2,4-dioxo-pyrimidine-1-carboxamide* (**15**). This compound was obtained as a white amorphous powder (46% yield). ^1^H-NMR (DMSO-*d*_6_) δ 4.54 (d, *J* = 5.6 Hz, 2H), 7.34–7.38 (m, 1H), 7.43–7.48 (m, 4H), 7.63–7.66 (m, 4H), 8.41 (br, 1H), 9.61 (t, *J* = 6.0 Hz, 1H), 12.29 (s, 1H); ^13^C-NMR (DMSO-*d*_6_) δ 44.3, 123.1, 123.5, 127.1, 127.2, 127.9, 128.5, 129.4, 137.9, 139.6, 140.4, 142.3, 150.6, 157.6; MS (ESI, *m*/*z*): 338 (M − H)^−^.

### 4.3. Synthesis of Uracil Derivatives ***16***–***17***

According to Method A, Scheme 2, 5-aminouracil (1270 mg, 10 mmol) and NaOH (600 mg, 15 mmol) were dissolved in H_2_O (20 mL) and then stirred at 0 °C for 10 min. Cbz-Cl (1.7 mL, 12 mmol) was added dropwise to the above mixture, and stirred overnight at room temperature. The resulting mixture was adjusted to pH 3 with dilute hydrochloric acid, and extracted with EtOAc. The organic layer was dried over anhydrous Na_2_SO_4_, and evaporated under reduced pressure to afford 5-(*N*-benzyloxycarbonyl)aminouracil.

5-(*N*-benzyloxycarbonyl)aminouracil (261.2 mg, 1 mmol), DMAP (6.1 mg, 0.05 mmol) and dried triethylamine (0.3 mL, 2 mmol) were dissolved in anhydrous pyridine (4.0 mL) and then stirred at 90 °C for 10 min under N_2_ protection. Phenylethyl isocyanate (220.8 mg, 1.5 mmol) were added dropwise, and the reaction mixture were stirred at 90 °C for 3 h. After cooling to room temperature, the reaction solution was concentrated under reduced pressure. The crude residue was purified by flash chromatography on silica gel to afford compound **16** (121.4 mg).

Compound **16** (98.2 mg, 0.24 mmol) was dissolved in anhydrous CH_2_Cl_2_ (4.0 mL), and stirred for 10 min at 0 °C. TMSI (0.1 mL, 0.48 mmol) was added dropwise in the above solution, and stirred for 2 h at the same temperature. CH_3_OH (1.0 mL) was added slowly to quench the reaction. After being stirred for 10 min, the reaction mixture was cooled and concentrated to remove the solvent. The residue was purified by flash chromatography on silica gel to afford compound **17** (21.6 mg).

*Benzyl* *(2,4-dioxo-1-{[(2-phenylethyl)amino]carbonyl}-1,2,3,4-tetrahydro-5-pyrimidinyl)carbamate (2H)-carboxamide* (**16**). This compound was obtained as a white amorphous powder in 30% yield. IR (ν_max_, cm^−1^): 2957, 2917, 2849, 1580, 1383, 1258, 1207; ^1^H-NMR (CDCl_3_) δ 2.90 (t, *J* = 7.1 Hz, 2H), 3.62–3.66 (m, 2H), 5.18 (s, 2H), 7.11 (s, 1H), 7.20 (s, 1H), 7.23–7.25 (m, 1H), 7.28–7.37 (m, 8H), 9.07–9.12 (br, 2H), 9.79(s, 1H); ^13^C-NMR (CDCl_3_) δ 35.4, 42.7, 67.6, 115.7, 123.6, 126.7, 127.1, 128.3, 128.5, 128.6, 128.7, 135.5, 138.3, 149.6, 149.8, 152.8, 159.4; MS (ESI, *m*/*z*): 407 (M − H)^−^.

*5-Amino-N-(2-phenylethyl)-2,4-dioxopyrimidine-1-carboxamide* (**17**). This compound was obtained as a white amorphous powder in 33% yield. IR (ν_max_, cm^−1^): 3337, 2955, 2921, 2851, 1723, 1613, 1580, 1462, 1378; ^1^H-NMR (DMSO-*d*_6_) δ 2.81–2.85 (m, 2H), 3.50–3.55 (m, 2H), 4.48 (s, 2H), 7.21–7.32 (m, 5H), 7.57 (s, 1H), 9.30–9.36 (m, 1H), 11.80 (br, 1H); ^13^C-NMR (DMSO-*d*_6_) *δ* 35.3, 42.2, 112.2, 124.5, 126.7, 128.9, 129.1, 139.4, 150.7, 151.0, 160.9; MS (ESI, *m*/*z*): 273 (M − H)^−^.

### 4.4. Synthesis of Compound ***18***

According to Method B, Scheme 2, 5-OTBS-uracil (96.9 mg, 0.4 mmol), DMAP (2.4 mg, 0.02 mmol) and anhydrous triethylamine (0.3 mL, 2 mmol) were dissolved in dry pyridine (4.0 mL). The reaction mixture was stirred for 10 min at 90 °C under N_2_ protection and then phenylethyl isocyanate (220.8 mg, 1.5 mmol) was added dropwise. After being stirred for 3 h at the same temperature, the reaction solution was cooled and concentrated under reduced pressure. The residue was purified by flash chromatography on silica gel to afford 5-OTBS-*N*-(2-phenylethyl)-2,4-dioxopyrimidine-1-carboxamide (25.2 mg). This reaction product (24.6 mg, 0.06 mmol) and TBAF 3H_2_O (39.9 mg, 0.13 mmol) were dissolved in THF (3.0 mL), and stirred for 30 min at 0 °C. After gradually warming to room temperature, the reaction solution was stirred for another 2 h. Solvent was evaporated under reduced pressure, and the crude residue was purified by flash chromatography on silica gel to afford compound *5-hydroxy-N-(2-phenylethyl)-2,4-dioxopyrimidine-1-carboxamide* (**18**) in 10% yield as a white amorphous powder. IR (ν_max_, cm^−1^): 2956, 2918, 2850, 1726, 1383, 1071; ^1^H-NMR (MeOD) δ 2.90 (t, *J* = 7.2 Hz, 2H), 3.61 (t, *J* = 7.2 Hz, 2H), 7.22–7.32 (m, 5H), 7.88 (s, 1H); MS (ESI, *m*/*z*): 274 (M − H)^−^.

### 4.5. General Procedure for the Synthesis of Uracil Derivatives ***19***–***21***

A solution of uracil derivative (0.5 mmol) in THF (3 mL) was added slowly to a suspension of NaH (60% in mineral oil, 30 mg) in anhydrous THF (5 mL) under a nitrogen atmosphere at −20 °C. After stirring for 5 min at −20 °C, a solution of the appropriate iodide (0.6 mmol) in THF (2 mL) was slowly added. The reaction mixture was stirred at 0 °C for 5 h and then quenched with saturated aqueous ammonium chloride solution (5 mL) followed by extraction with EtOAc (3 × 5 mL). The combined organic layers were dried over anhydrous Na_2_SO_4_ and filtered. The concentrated residue was purified by flash chromatography on silica gel to afford compounds **19**–**21**.

*1-Heptyl-2,4-dioxopyrimidine* (**19**). This compound was obtained in 44% yield as a white amorphous powder. IR (ν_max_, cm^−1^): 2920 (N-H), 2852 (N-H), 1667 (C=O), 1461; ^1^H-NMR (CDCl_3_) δ 0.89 (t, *J* = 6.8 Hz, 3H), 1.28–1.32 (m, 8H), 1.67–1.71 (m, 2H), 3.73 (t, *J* = 7.6 Hz, 2H), 5.71 (d, *J* = 7.6 Hz, 1H), 7.16 (d, *J* = 7.6 Hz, 1H), 9.56 (s, 1H); ^13^C-NMR (CDCl_3_) δ 14.0, 22.6, 26.4, 29.0, 29.1, 31.7, 48.9, 102.1, 144.5, 151.0, 164.0; MS (ESI, *m*/*z*): 209 (M − H)^−^. Anal. Calcd. for C_11_H_18_N_2_O_2_: C 62.83; H 8.63; N 13.32. Found: C 62.59; H 8.64; N 13.30. This is a known compound, although the detailed MS, ^1^H- and ^13^C-NMR data are not reported in the previous literature.

*5-Fluoro-1-heptyl-2,4-dioxopyrimidine* (**20**). This compound was obtained as a white amorphous powder in 31% yield. IR (*ν*_max_, cm^−1^): 2920 (N-H), 2852 (N-H), 1667 (C=O), 1461; ^1^H-NMR (CDCl_3_) δ 0.88 (t, *J* = 6.8 Hz, 3H), 1.26–1.33 (m, 8H), 1.67–1.69 (m, 2H), 3.70 (t, *J* = 7.6 Hz, 2H), 7.23 (d, *J* = 5.2 Hz, 1H), 9.33 (s, 1H); ^13^C-NMR (CDCl_3_) *δ* 14.0, 22.6, 26.3, 28.9, 29.0, 31.7, 49.1, 128.7, 139.2, 149.3, 157.3; MS (ESI, *m*/*z*): 227 (M − H)^−^. Anal. Calcd for C_11_H_17_FN_2_O_2_: C 57.88; H 7.51; N 12.27. Found: C 58.05; H 7.52; N 12.29.

*2-[5-Fluoro-2,4-dioxopyrimidinyl]-N-hexylacetamide* (**21**). This compound was obtained in 46% yield as a white amorphous powder. Detailed MS and ^1^H-NMR results matched the literature values [23].

### 4.6. Enzyme Activity Assay

The inhibition effects of uracil derivatives toward recombinant rat FAAH (rFAAH) were assessed by enzyme activity assay [24]. HEK293-rFAAH cells were harvested, washed with PBS, sonicated in 20 mM Tris-HCl (pH 7.5) containing 0.32 M sucrose, and centrifuged at 800× *g* for 15 min at 4 °C. The supernatants were collected and protein concentrations were measured by BCA protein assay kit (Pierce, Shanghai, China). FAAH proteins (30 μg) were incubated at 37 °C for 30 min in Tris buffer (50 mM, pH = 8.0) containing fatty acid-free BSA (0.05%) and 25 μM AEA (Sigma-Aldrich, Shanghai, China) as substrate. The above reactions were stopped by adding 200 μL methanol containing 1 nmol heptadecanoic acid (17:0 FFA) as internal standard. The reaction mixture was extracted with 4 mL chloroform and then vortex for 1 min. Lipid layer was separated by centrifugation at 3000× *g* for 10 min and then transferred to a clean 10 mL V-bottom glass tube and dried under nitrogen (N_2_) flow. After redissolved with 1 mL CHCl_3_, the lipid solution went through the solid-phase extraction, and was eluted by methanol/chloroform (*v*/*v*, 1/9). The elution containing FAEs and FFAs was dried under N_2_, and reconstituted in 100 μL methanol for HPLC-MS/MS analysis.

Samples were detected by an ABI 3200 Q-Trap mass spectrometer (Applied Biosystems/MDS Sciex) equipped with 1100-LC system (Agilent, Shanghai, China). The mobile phase for FFAs detection was consisted of MeOH and ultra-pure H_2_O (pH = 7.4, each containing 0.25% acetic acid and 5 mmol/L ammonium acetate), and the elution condition was 95% methanol keeping for 3 min at a flow rate of 1.0 mL·min^−^^1^. The column temperature was kept at 40 °C. Ion detection was monitored by ESI negative mode. The molecular ions were monitored at *m*/*z* 303 for AA, and *m*/*z* 269 for C17:0 FFA. Inhibition curves and IC_50_ values of each compounds was calculated by GraphPad Prism (version 5.01, GraphPad Software Inc., San Diego, CA, USA).

## 5. Conclusions

According to the crystal structure of protein rFAAH-MAFP (PDB: 1MT5), the catalytic core of FAAH contains a series of channels and cavities including a cytosolic port (CP) which interacts with the hydrophilic fragments and a acyl-chain binding pocket (ACBP) that may allow for the exit of the acyl chain to support the catalytic reaction. Herein, we used the uracil ring as a novel hydrophilic product scaffold, and different aromatic substituent groups to replace the acyl side chain. Urea bonds were formed to connect these two fragments, and thus a series of structurally related substituted 2,4-dioxopyrimidine-1-carboxamides were designed and synthesized according to Scheme 1, Scheme 2 and Scheme 3.

In the present study, we have discovered and identified uracil derivatives as a novel class of potent FAAH inhibitors with IC_50_ values in the nanomolar or low-micromolar range. Initial SAR analysis indicated that an entirely conjugated 2,4-dioxopyrimidine-1-carboxamide scaffold was necessary for FAAH inhibition. Introduction of a 4-biphenylmetyhl moiety as the *N*-acyl chain was favorable for activity, leading to a highly potent inhibitor. Although replacement of a 2-phenylethyl side chain decreased the inhibitory potency, electron-withdrawing substituents in the 5-position of the uracil ring afforded two novel double digit nanomolar FAAH inhibitors. An electron-withdrawing moiety rather than an electron-donating group at position 5 of the 2,4-dioxopyrimidine is beneficial for approaching potent FAAH inhibition. However, further investigation is necessary to elucidate the exact mechanism of action of this class of inhibitors, which should be useful to clarify the structure-activity relationships to enhance FAAH affinity and selectivity.

## Figures and Tables

**Table 1:** FAAH inhibitory activity of compounds **1**–**21** and URB597.

Compound	IC_50_ of FAAH Inhibition (μM)	Compound	IC_50_ of FAAH Inhibition (μM)
**1**	0.11 ± 0.03	**12**	0.81 ± 0.10
**2**	>10	**13**	1.21 ± 0.22
**3**	>10	**14**	0.053 ± 0.006
**4**	0.86 ± 0.08	**15**	0.10 ± 0.01
**5**	0.25 ± 0.03	**16**	0.12 ± 0.03
**6**	1.27 ± 0.21	**17**	1.33 ± 0.13
**7**	0.30 ± 0.07	**18**	0.54 ± 0.07
**8**	1.07 ± 0.14	**19**	>100
**9**	0.531 ± 0.08	**20**	>100
**10**	0.098 ± 0.01	**21**	>100
**11**	0.021 ± 0.004	URB597	0.035 ± 0.008
